# Trade-Offs between Geographic Scale, Cost, and Infrastructure Requirements for Fully Renewable Electricity in Europe

**DOI:** 10.1016/j.joule.2020.07.018

**Published:** 2020-09-16

**Authors:** Tim Tröndle, Johan Lilliestam, Stefano Marelli, Stefan Pfenninger

**Affiliations:** 1 Institute for Advanced Sustainability Studies, Berliner Straße 130, 14467 Potsdam, Germany; 2 Institute for Environmental Decisions, ETH Zürich, Universitätstrasse 16, 8092 Zürich, Switzerland; 3 Faculty of Economics and Social Sciences, University of Potsdam, August-Bebel-Strasse 89, 14482 Potsdam, Germany; 4 Chair of Risk, Safety and Uncertainty Quantification, ETH Zürich, Stefano-Franscini-Platz 5, 8093 Zürich, Switzerland

**Keywords:** energy, decarbonization, self-sufficiency, cooperation, trade, transmission, regional equity, land use, acceptance, flexibility

## Abstract

The European potential for renewable electricity is sufficient to enable fully renewable supply on different scales, from self-sufficient, subnational regions to an interconnected continent. We not only show that a continental-scale system is the cheapest, but also that systems on the national scale and below are possible at cost penalties of 20% or less. Transmission is key to low cost, but it is not necessary to vastly expand the transmission system. When electricity is transmitted only to balance fluctuations, the transmission grid size is comparable to today’s, albeit with expanded cross-border capacities. The largest differences across scales concern land use and thus social acceptance: in the continental system, generation capacity is concentrated on the European periphery, where the best resources are. Regional systems, in contrast, have more dispersed generation. The key trade-off is therefore not between geographic scale and cost, but between scale and the spatial distribution of required generation and transmission infrastructure.

## Highlights

• Continent-wide renewable electricity supply is cheapest but needs large grid expansion
• Small-scale supply has only 20% higher cost but needs more generation infrastructure
• On all supply scales, expanding cross-border transmission is necessary for low cost
• Key trade-off is between geographic scale and infrastructure requirements

## Context & Scale

Transitioning to fully renewable electricity in Europe requires at least four to five times more solar and wind generation capacity than the continent hosts today. Where exactly all this infrastructure should be built is controversial. Options range from a continental-scale system with access to Europe’s best renewable resources to small-scale locally sufficient systems.

We analyze how system cost and infrastructure requirements change with system scale. We find that continental supply has the lowest cost and requires less generation infrastructure than small-scale supply. However, it requires a large expansion of the transmission grid. While supply scale is not the main cost driver, the scale at which balancing of variable renewable generation takes place is critical for cost: continental-scale balancing leads to low-cost electricity. The key trade-off for European decision-makers is therefore not just between scale and cost, but between supply scale and infrastructure requirements.

## Graphical Abstract

**Figure:**
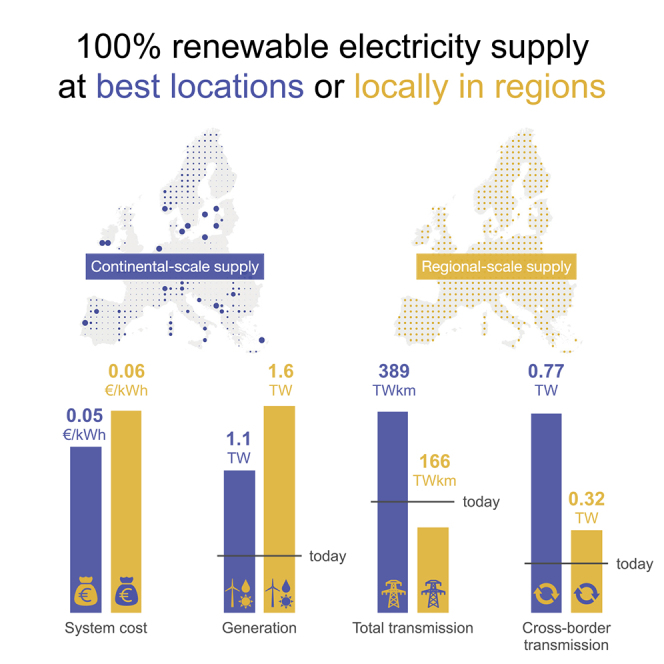

## Teaser

We show that fully renewable electricity supply in Europe is cheapest when supply happens on the continental scale, but that national or subnational supply is possible at cost penalties of 20% or less. Such low-cost electricity requires continental-scale balancing of renewable fluctuations and an expansion of cross-border transmission capacities, but not a large transmission grid overall. Infrastructure requirements vary strongly between supply on different scales, and thus, the key trade-off is between infrastructure requirements and scale.

## Introduction

To fulfill its commitment under the Paris climate agreement, Europe must eliminate electricity sector emissions. For this, future electricity supply will be based largely, or entirely, on renewable sources.^1^ Although ambitious, this is possible because solar and wind generation technologies are mature,^2^^,^^3^ their generation potential is sufficient,^4^, ^5^, ^6^, ^7^, ^8^ various options are available to balance variable renewable generation on time scales ranging from hours to years,^9^, ^10^, ^11^ and systems relying on them can have similar cost as today’s system.^2^^,^^12^, ^13^, ^14^ As renewables are abundantly available across the continent, very different kinds of future electricity system designs are possible, from a Europe-wide electricity grid sharing generation resources among all countries, to myriads of locally self-sufficient units, either disconnected or with limited interconnection, as well as combinations of these two extremes.^15^^,^^16^ Here, we investigate the impact on cost and cost-optimized system design of decarbonizing Europe’s electricity supply using renewables on different geographic scales and with different degrees of self-sufficiency.

The European energy transition is highly politicized, and citizen engagement is one of its historical drivers.^17^ Ideas of decentralization, energy democracy, and local control have great appeal to many citizens and decision-makers, leading to calls for regionally self-sufficient systems based on local resources.^18^, ^19^, ^20^, ^21^ In such systems, generation variability would be balanced locally using electricity storage and locally available dispatchable resources, without the need for new transmission infrastructure. The annual renewable potential for local self-sufficiency is large enough in most of Europe,^6^ but it is not known how balancing of fluctuations on smaller timescales affects cost and the system design in different regions.

Others, in contrast, point to the cost reduction potential of a continent-spanning supply system, stemming from sharing the best renewable resources and from relying on stochastic smoothing of supply fluctuations through large grids, while making efficient use of dispatchable resources regardless of where they are located.^10^^,^^22^, ^23^, ^24^ Indeed, previous research has shown that these effects make a continent-spanning renewables-based system cheaper than smaller systems. A previous study found that total system cost rises by 12% when renewable electricity cannot be generated anywhere on the continent, but only within national borders.^25^ If not only generation, but also the balancing of fluctuations happens within national borders and countries do not trade, cost rises to 122% of the continental-scale case.^25^ Another study found slightly higher cost of self-sufficient countries of 130%.^11^ The cost penalty has been found to be only 10% when, instead of individual countries, small groups of countries are fully self-sufficient.^14^ These findings indicate that cost indeed decreases with geographic scale.

No previous study has assessed the effect for regions below the national level. In addition, most of the previous work changed system designs along two dimensions simultaneously: supply scale and balancing scale. We define supply scale as the geographic extent across which systems are net self-sufficient over a whole year, and balancing scale as the geographic extent across which systems balance fluctuations from renewable supply (see Experimental Procedures). In a system with national supply and continental balancing, for example, each country in Europe generates sufficient electricity annually to satisfy national demand, but all countries can still trade within a year to balance renewable fluctuations. By varying both simultaneously, past work was unable to attribute observed effects to their root causes, for example to access to better renewable resources, access to dispatchable resources, or stochastic smoothing in large grids. It is, furthermore, not clear whether self-sufficiency is necessarily more expensive in smaller systems than larger systems: possibly, the cost penalty of smaller-scale self-sufficiency can be reduced or eliminated through appropriate system design.

Here, we model fully renewable European electricity systems and vary supply scale and balancing scale independently across continental, national, and regional (subnational) levels (see Figure S1). This allows us, for example, to consider net self-sufficient regional electricity systems, which trade with the entire continent to balance their local supply. We use a cost-minimizing linear programming model that considers solar, wind, hydropower, and bioenergy, based on the *Calliope* framework.^26^ It is spatially resolved to first-level administrative divisions (497 subnational regions) of the EU-28, Norway, Switzerland, and Western Balkan countries and runs 1 year of recorded data at a 4-h temporal resolution (see Experimental Procedures for a detailed description of the model). We also conduct a sensitivity analysis based on a multi-fidelity sparse polynomial chaos expansion of the original model, permitting us to explore a large range of uncertainties despite the main model being computationally difficult to solve (see Experimental Procedures).

### System Scale Drives System Cost

**Figure 1:**
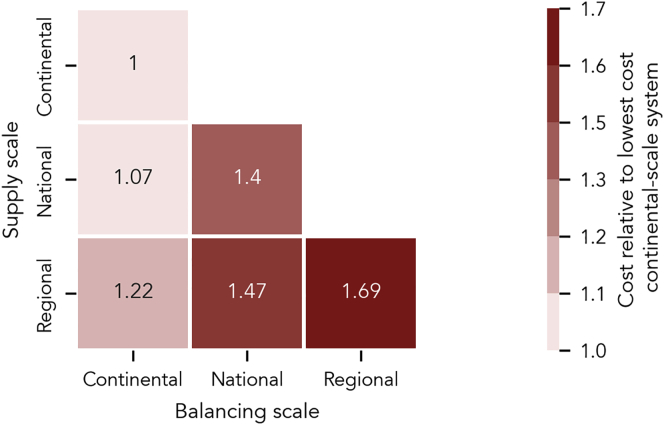
System Cost of Six Electricity Systems in Europe with Supply and Balancing on Three Different Scales, Relative to Lowest Cost, Continental-Scale System In all six cases, net supply is generated (x axis) and balanced (y axis) either on the entire continent, within countries, or within subnational regions. Systems on the diagonal are entirely continental, national, or regional scale with no trade between units on the respective scale. Systems below the diagonal are net self-sufficient (zero trade balance over a whole year) on their respective supply scale with trade for balancing between units on this scale.

We first assess the impact of three different system scales on total system cost and find that there is a strong trade-off between balancing scale and cost, but only a weak trade-off between supply scale and cost (Figure 1). As expected, system cost increases when either supply or balancing scale decreases. System scale matters mainly because interconnecting a wider geographic area allows dispatchable generation options such as hydropower and bioenergy to percolate across the entire European system, lowering the total balancing cost. Furthermore, a geographically larger supply system covers a greater geographic area and, thus, a greater variety of wind and solar resources, including higher-quality ones out of reach in smaller-scale cases. For an entirely continental-scale system, in which supply and balancing of supply spans the entire continent, the cost-optimized system configuration corresponds to about 0.05 EUR per kWh of electricity demand, which is comparable to today’s cost. Limiting the net supply options to those available within countries or regions increases cost relative to the continental case to 107% and 122%. When we additionally decrease the balancing scale to the national level, reaching 33 isolated national systems, the costs sharply increase to 140% (national supply and balancing) and 147% (regional supply, national balancing). Balancing of supply on the lowest scale, i.e., disabling electricity trade between the 497 now-isolated subnational regions in Europe, raises cost to 169% of the continental case (Figure 1). The main cost driver of these small, isolated systems is the limited access to regionally concentrated balancing options, such as hydropower, but also the impossibility to share the best wind and solar resources among regions. Allowing for net imports hardly affects cost (see Experimental Procedure S1 and Figure S2), again emphasizing that balancing rather than supply resource quality is the main source of cost reduction in larger systems. Therefore, local electricity generation must not be substantially more expensive than a continental-scale supply, if such regionally net self-sufficient units are interconnected through the transmission grid to balance the fluctuating renewable generation.

**Figure 2:**
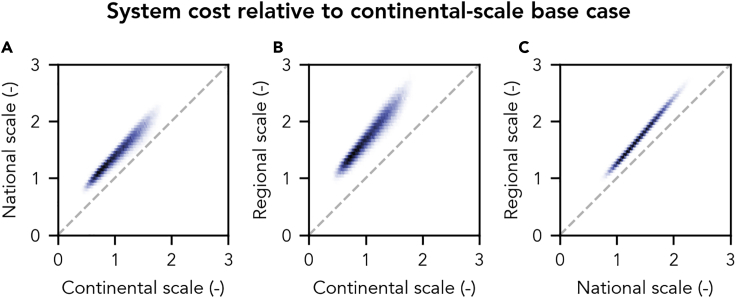
Uncertainty of System Cost in Electricity Systems for which Supply Scale Equals Balancing Scale Considering Uncertainty in Twelve Input Parameters (A–C) Bivariate histograms of the joint distributions of variability within entirely continental- and national- (A), entirely continental- and regional- (B), and entirely national- and regional-scale systems (C). For each scale, 100,000 samples are obtained with surrogate models fitted to the main optimization model. Darker colors indicate more occurrences of values within these 100,000 samples. System cost is normalized by the cost of the continental base case (Figure 1). For a detailed description of all assessed input uncertainty, see Table S1.

Despite large uncertainty about technology and total system cost, the general relationship between system scale and cost is robust. When varying technology cost assumptions using surrogate models fitted to the main optimization model (through multi-fidelity sparse polynomial chaos expansion, see Experimental Procedures), the resulting cost differences between system scales vary, but the entirely continental-scale system is always cheaper than the entirely national-scale system, which in turn is always cheaper than the entirely regional-scale one (Figure 2).

### System Scale Drives Technology Deployment

**Figure 3:**
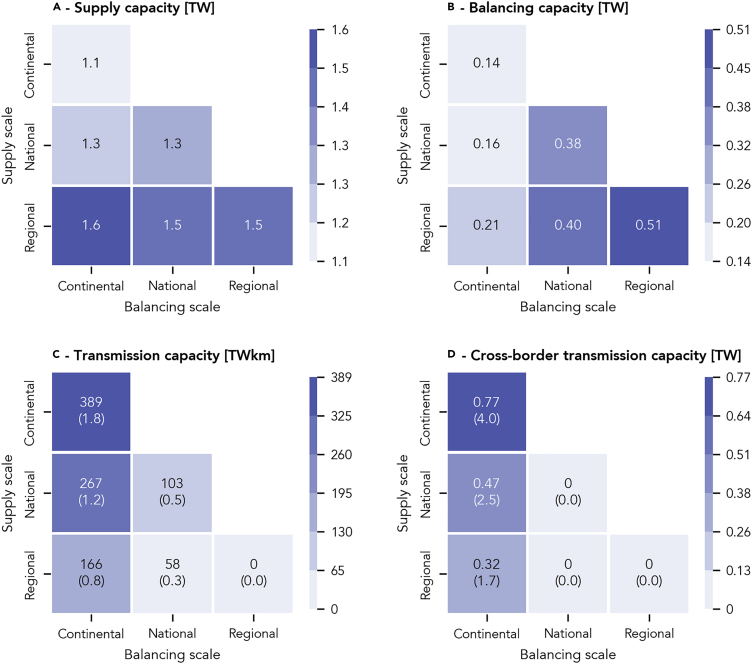
Cost-Optimized Supply, Balancing, and Transmission Capacities of Six Electricity Systems with Supply and Balancing on Three Different Scales (A) Total supply capacity comprising wind, solar, and hydro power. (B) Total balancing capacity comprising battery, hydrogen, and pumped hydro storage and bioenergy capacity. (C) Transmission capacity between all regions. Numbers in brackets are relative to today’s capacity of 215 TWkm (see Experimental Procedure S2 on how we determine today’s capacity). (D) Cross-border transmission capacity. Numbers in brackets are relative to today’s capacity of 0.190 TW (see Experimental Procedure S2 on how we determine today’s capacity). See Tables S2 and S3 and Figure S3 for all data. See Figure S4 for generation data resolved at national levels.

Geographic scale strongly affects the deployment of generation, transmission, and balancing technologies. Systems with supply on small scales require more generation capacity than larger-scale ones (Figure 3A), as they must often rely on poorer local renewable resources to meet their annual demand. Regional-scale supply, thus, requires the most installed wind, solar, and hydro power capacity and exceeds the capacity of a continental-scale system by 40%. The additional generation capacity investment needed in systems with smaller supply scale is one driver of their higher system cost.

The scale on which renewable fluctuations are balanced drives the required levels of storage and flexible generation capacities (Figure 3B). With smaller geographic extents, the correlations of fluctuations of solar and especially wind power rise, and with it, total fluctuations increase. Locally, these fluctuations can only be handled by either electricity storage or by flexible generation from bioenergy. Continental-scale balancing, in contrast, can exploit wind patterns across Europe on multiple timescales, thereby strongly reducing aggregated wind fleet fluctuations and the need for additional balancing, which has been demonstrated previously.^10^^,^^27^ Therefore, regional-scale balancing requires 140% more balancing capacity than in the continental case, which is an important driver of its high system cost. The supply scale also drives balancing capacities because restrictions on the locations of supply technologies limit the extent to which continental-scale balancing can be exploited. In comparison to balancing scale, its impact on balancing capacities is minor.

Systems on larger geographic scales are stronger connected and thus require larger transmission systems. Both scales, supply and balancing, increase the requirements for transmission capacities (Figure 3C). On large supply scales, electricity is transmitted mainly unidirectionally from locations with best renewable resources to demand centers. On large balancing scales, electricity is bidirectionally shifted between subnational regions. However, continental-scale balancing requires less transmission capacity than continental-scale supply. As long as supply is regional or national, continental-scale balancing requires transmission capacities in the order of today’s capacity. When supply is continental, capacity requirements rise to 390 TWkm, roughly two times the current European transmission system.

Continental-scale balancing requires a layout of the transmission system that deviates from today’s. While its total size (in TWkm) is roughly similar to today’s as long as supply is regional or national, it requires roughly a doubling of cross-border capacities (Figure 3D). Supply on larger scales has higher needs for cross-border capacities, rising to roughly four times of what exists today. To exploit the benefits of continental-scale balancing, cross-border capacities must be expanded significantly.

**Figure 4:**
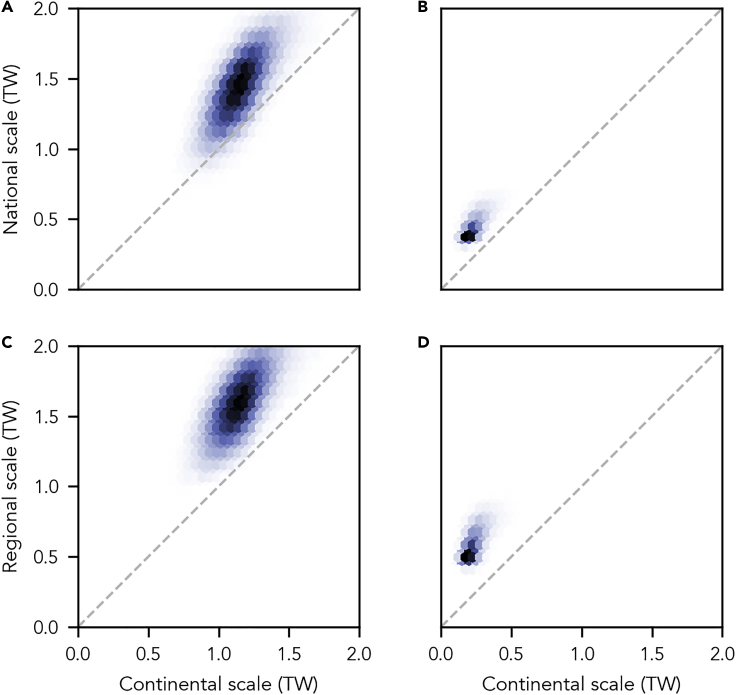
Uncertainty of Installed Capacities in Electricity Systems for which Supply Scale Equals Balancing Scale Considering Uncertainty in Twelve Input Parameters (A–D) Each panel shows a bivariate histogram of the joint distribution of variability within entirely continental- and national- (A and B) and entirely continental- and regional-scale systems (C and D). Darker colors indicate more occurrences of values within 100,000 samples of each surrogate model. (A and C) Variability of total supply capacity comprising wind, solar, and hydro capacities. (B and D) Variability of total balancing capacity comprising battery, hydrogen, and pumped hydro storage and bioenergy capacities. For a detailed description of all assessed input uncertainty, see Table S1.

The differences in system structure and capacity deployment between continental-, national-, and regional-scale systems are unaffected by input uncertainties (Figure 4 and Table S1). Cost-optimized, entirely national- and regional-scale systems always have more combined balancing capacity and almost always have more total supply capacity than cost-optimized entirely continental-scale systems.

### System Scale Drives Spatial Distribution of Technology Deployment

**Figure 5:**
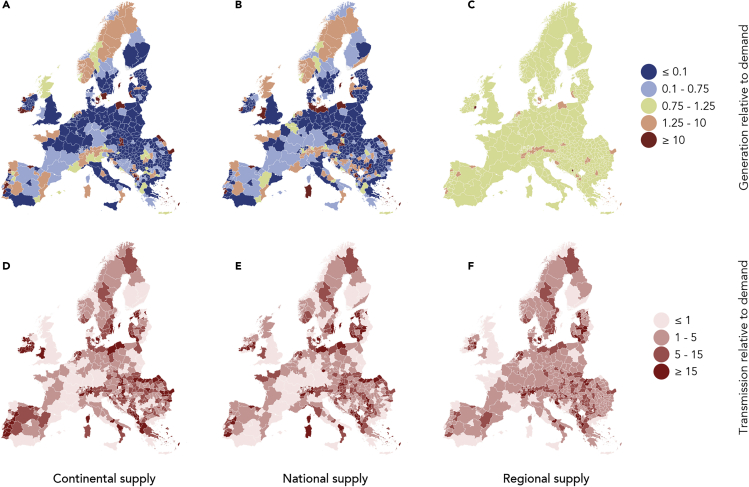
Spatial Distribution of Generation and Transmission Relative to Demand for Each Region in Europe in Three Systems with Continental-Scale Balancing All values are annual sums and relative to annual local electricity demand. (A–C) Electricity generated using a continental-scale (A), national-scale (B), and regional-scale (C) supply. (D–F) Electricity transmitted using a continental-scale (D), national-scale (E), and regional-scale (F) supply.

Expanding renewables using a continental-scale supply is the least-cost option, but doing so means that generation and transmission are unequally distributed across Europe. In the continental-scale supply system, peripheral regions generate electricity for the central parts of the continent. For example, Ireland, Lithuania, Estonia, and Albania generate more than 400% of their own electricity demand, requiring a land area four times larger than necessary for their own needs. This effect is even more pronounced in single subnational regions: several Irish counties facing the Atlantic ocean—such as Mayo, Kerry, or Cork—the Baltic Sea islands Gotland and Saaremaa, or Tulcea at the Romanian Black Sea coast generate over 50 times their own demand (Figure 5A) by fully exploiting their technical generation potential almost exclusively for export. In contrast, other regions and countries rely strongly, or sometimes entirely, on imports: Belgium, Czech Republic, and Germany, for example, produce less than 10% of their own electricity demand. The spatial distribution is sensitive to cost and resource assumptions, as minimization moves the bulk of generation to locations with best conditions, even if the difference to the second best location is minor; yet, the finding that generation capacity is centralized in the continental-scale supply system is robust.

To enable this trade, a continent-spanning transmission system is needed. In extreme cases, over 250 times the local demand is transferred through a region, for example in southern Ireland, where electricity from coastal wind farms is transferred to Wales and then onward to England and central Europe (Figure 5D). Similar effects are found in peripheral regions in the Baltic Sea region, Portugal, Romania, and the Western Balkans. Large amounts of electricity are exported from and through these regions, requiring correspondingly large transmission capacities from which most citizens do not benefit directly, raising questions of the social acceptability of such schemes.

Generation and transmission are more homogeneously distributed for national-scale (Figures 5B and 5E) and regional-scale (Figures 5C and 5F) supply. In the national case, most generation hot spots remain, but their extent is smaller as they only supply national, not continental, demand. In the regional case, the generation pattern changes radically as each region generates electricity to cover its own demand only. Some regions have slightly higher generation capacities that are used to compensate for transmission losses between regions. The transmission pattern does not change as strongly because transmission remains crucial to balance renewable fluctuations. However, transmission hot spots are much less pronounced as supply is distributed more homogeneously, and the grid is only used for balancing, but not for bulk supply. National- and especially regional-scale supply thus lead to higher regional equity in terms of generation and transmission infrastructure.

**Figure 6:**
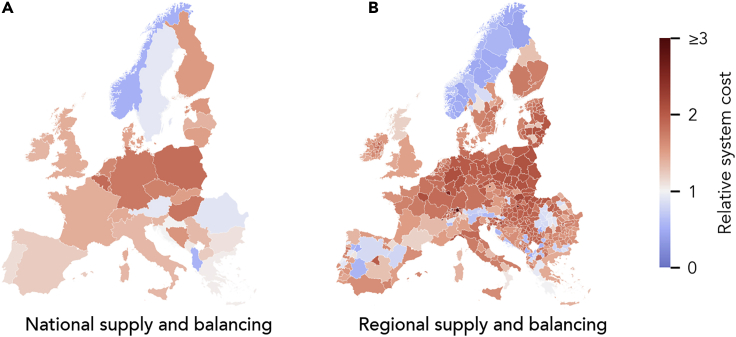
Spatial Distribution of System Cost Normalized by Demand for Entirely National- and Regional-Scale Systems, Relative to the European Average of the Least-Cost, Entirely Continental-Scale Case Each panel shows the Europe-wide relative system cost beneath the panel heading. (A) Relative system cost for each country in the entirely national-scale system without any exchange of electricity between countries. (B) Relative system cost for each region in the entirely regional-scale system without any exchange of electricity between regions.

In entirely small-scale systems, when regional electricity systems are fully self-sufficient and thus isolated, regional generation infrastructure equity is highest, but system cost varies strongly between regions, depending on the available renewable resources (Figure 6B). About 12% of regions (and a few countries) have cost below the European average of the continental-scale baseline (blue areas in Figure 6). These regions cover 35% or more of their peak demand with low-cost hydropower, which we assume is fully depreciated, with only O&M cost in our model (see Experimental Procedure S3).

Another 20% of regions have cost at least twice those of the least-cost European average. These expensive, mainly city regions like Geneva, Prague, Budapest, and Bucharest, have low or no potential for wind power, and thus, their main source of electricity is the sun. As solar generation has strong seasonal fluctuations in Europe, these regions require more flexible generation from bioenergy or hydropower or long-term electricity storage to cover winter demand. Where the potentials for wind are low, the cost of providing flexibility, and thus system cost, is particularly high.

### Technology Cost Drives Relative Attractiveness of Scales

**Figure 7:**
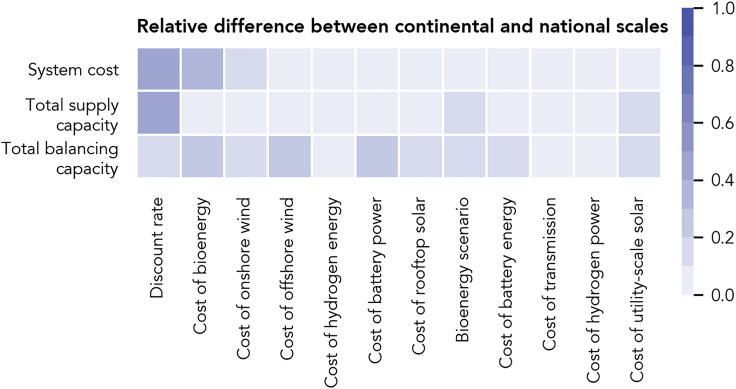
Total Sobol’ Indices for Combinations of All Considered Input Uncertainties and Output Differences between Entirely Continental- and National-Scale Systems The total Sobol’ indices determine the magnitude with which the variability of one model input explains the variability of one model output. The x axis shows the twelve input parameters included in the uncertainty analysis, sorted by their impact on system cost. The y axis shows the model-wide result variables for which continental to national-scale differences are compared. See Table S1 for a detailed compilation of all considered input uncertainty. See Figures S5–S7 for total Sobol’ indices, first-order Sobol’ indices, and the difference thereof, for all relevant model outputs on all three scales.

Above (Figures 2 and 4), we showed how the qualitative differences between scales are robust to input uncertainty. Nevertheless, uncertainties in model inputs affect both the cost and system design in all cases. Through a global sensitivity analysis, we find that the uncertainties of three input parameters explain by far most of the uncertainty in cost and design differences between entirely continental- and national-scale systems: discount rate, overnight cost of bioenergy, and overnight cost of onshore wind power (Figure 7). Uncertainties regarding the other parameters impact our results less strongly. Because we use a different sensitivity analysis method on the regional scale compared with the national and continental scales (see Experimental Procedures) we cannot analyze sensitivities of scale differences involving the regional scale and therefore analyze results on the regional scale only in absolute terms (see Figures S5–S7 for sensitivities of absolute and relative values on all three scales).

Entirely national-scale systems become relatively more cost attractive when discount rate or overnight cost of onshore wind power is high. Higher discount rates increase the cost of transmission lines particularly strong due to their long lifetime. Similarly, high wind power cost causes lower deployment of wind capacity and therefore reduced usefulness of transmission lines. Consequently, in a high discount rate and wind power cost setting, a cost-optimized continental-scale system contains less transmission capacity and resembles national-scale systems more closely. Cost and design differences between the continental and national cases decrease. In contrast, when cost of bioenergy is high, national-scale systems become relatively less cost attractive, as seasonal fluctuations are more pronounced on the national scale, and generally bioenergy is the least-cost option to balance them. Increases in cost of bioenergy, thus, increases total system cost of the national-scale systems more strongly than cost of the continental case. All three parameters do not change the qualitative relationship between geographic scale, cost, and design, but they do change the magnitude with which costs and designs differ across scales.

## Discussion and Conclusions

We show that European renewable electricity systems on larger geographic scales always have lower cost, especially as they have more flexibility options available to balance fluctuating supply, but also that small-scale supply can be designed to have only small cost penalties by allowing continental-scale balancing. Regionally self-sufficient systems have high cost mainly because they cannot access the grid and dispatchable resources outside their own territory for balancing. This is why regional net self-sufficient systems, which supply their own electricity but trade with other subnational regions and countries for balancing, have low cost: the fluctuations are smoothed via the grid, and they can access dispatchable resources outside their own territory. Hence, an electricity system with continental-scale supply and balancing is the least-cost option for fully renewable power in Europe, but regional-scale supply can also be low cost, if allowing for continental-scale balancing.

This means that large and small systems need not differ much in cost, but they differ strongly in system structure, and the infrastructure requirements make land use and the physical appearance important trade-offs to geographic scale. Using the transmission grid for continental-scale supply, i.e., transmitting electricity from Europe’s best resources to demand centers, has the lowest cost but requires large transmission capacities of up to two times today’s transmission grid. Using the transmission grid for continental-scale balancing of net self-sufficient regional supply, in contrast, requires much less transmission capacity—roughly the size of today’s transmission system albeit with twice the cross-border capacities. Regional-scale supply, however, has much higher generation capacity needs than continental-scale supply, and all of this generation is necessarily located near demand centers and cities, where pressure on land is already high. Hence, by scaling the European electricity system on the supply and balancing side independently, very different renewable electricity system designs are possible. While systems with continental-scale balancing all have similar costs, they differ strongly regarding how much and where transmission and generation assets are needed.

By applying a multi-fidelity sparse polynomial chaos expansion to our high-resolution electricity system model, we show that the findings are unaffected by the input parameter uncertainty we consider. However, the cost outlook for specific technologies influences the relative cost differences between larger-scale and smaller-scale systems. Two aspects that our analysis does not consider may make small-scale systems more cost attractive. First, additional flexibility deriving from electrifying the heat and mobility sectors could reduce the cost of flexibility,^28^ which in our model is particularly high on small scales. Second, ancillary services that must be provided locally could limit the otherwise unrestricted spatial deployment on the continental scale. The first aspect may decrease cost differences on the balancing scale only, while the second aspect may decrease cost differences on both the supply and the balancing scale. For a detailed discussion of these effects, see Experimental Procedure S4.

Extending previous studies,^11^^,^^25^^,^^29^ we confirm that fully renewable electricity supply in Europe does not necessarily require vastly increased transmission capacities, contradicting recent statements and views.^30^ By allowing systems to balance regional-scale supply within a continental grid, their cost penalty can be reduced to 20% above the least-cost, continental-scale system but without the need for large transmission expansion. We further show that a fully renewable electricity system is possible not only in continent-spanning^29^^,^^31^^,^^32^ and national^11^^,^^25^ designs, as previous work has shown, but also on the regional scales, and we show why cost and design differences appear across the different scales. While cost and total generation capacity are higher on smaller scales, they are likely not so high as to be economically infeasible, and these higher costs allow system operators to avoid transmission capacity expansion. The cost differences can be expected to be even smaller should the heat and transport sectors be coupled tightly with the electricity sector.^28^^,^^33^ Our results show how system cost of fully renewable electricity systems depend strongly on the balancing scale, but not as much on the supply scale, and that transmission needs can be traded off against generation capacity requirements. Thus, we show that very different system designs are possible, from the very small and regional to the very large and continental. It is important that policymakers and societies decide which type of system they find most attractive, in the knowledge that only one or the other can be built and that countries and citizens must accept either generation or transmission infrastructure for a transition to a fully renewable future in Europe to be feasible.

## Experimental Procedures

### Resource Availability

#### Lead Contact

Please contact the Lead Contact, Tim Tröndle (tim.troendle@iass-potsdam.de) for information related to the data and code described in the following Experimental Procedures section.

#### Materials Availability

No materials were used in this study.

#### Data and Code Availability

The datasets generated during this study are available on Zenodo https://doi.org/10.5281/zenodo.3950308.

The model code and all analysis steps are publicly available as a reproducible Snakemake^34^ workflow on Zenodo https://doi.org/10.5281/zenodo.3949794 and https://doi.org/10.5281/zenodo.3950775.

### Model Overview

We model a possible future European electricity system as a set of network nodes and power flows between the nodes, with each node representing a regional administrative unit in Europe. We consider the deployment of renewable electricity supply and storage technologies at each node, and the deployment of transmission links between nodes, but disregard subordinate network nodes and power flows on the distribution system. We do not consider current legacy generation capacities or the current topology of the transmission system and, thus, our model represents a greenfield system. We use this approach in order to understand the effect of system scale and size irrespective of the influence of legacy generation.

Using the Calliope model framework,^26^ we build a linear programming model that simultaneously optimizes electricity system design and operation for a single weather year, 2016, with a temporal resolution of 4 h (see Experimental Procedure S5 for a discussion of the choice of the resolution). We choose a single year to reduce problem size, but we test this choice by also modeling the weather years 2007–2016 in a sensitivity analysis (see below). The objective function of the model is to find the design with the lowest total system cost. An electricity system design is defined by a set of supply capacities at each node, storage capacities at each node, and transmission capacities between all nodes. All system designs fulfilling Kirchhoff’s law, the technical constraints of all possible technology components, and political constraints (see below) are possible. We assume that electricity generation from photovoltaics (PVs), on- and offshore wind, and hydropower plants can be curtailed, i.e., the model can decide to lower actual generation at a certain point in time from the maximum generation given by the capacity factor time series described below.

The following text describes the modeling choices, applied datasets, and assumptions made.

### Geographic Scope and Transmission Grid

The study area comprises all countries represented by member organizations in the ENTSO-E: the EU-28, Norway, Switzerland, and Western Balkan countries. We exclude Iceland, which is electricity autarkic, Cyprus, which is not directly connected to the rest of the study area, and Malta, for which insufficient data are available. We divide the study area into 497 regional administrative units,^6^ each of which is considered to be a transmission network node. We model the transmission grid as direct net transfer capacities between network nodes, i.e., we consider net power flows on the shortest distances between nodes only, and assume the distribution network within each node is able to handle distribution load. We allow transmission capacities between regional administrative units sharing a land border. We use currently existing sea connections and those that are currently under construction to connect regions that do not share a land border.^35^ We furthermore connect the islands Hiiu and Saare to the Estonian mainland, resulting in a fully connected electricity network graph as visible in Figure S5.

### System Scale

We use two types of geographic scale as the basis for our system layouts. First, the scale of the electricity supply, and second, the scale of balancing of supply. Electricity supply on the continental, national, or regional scale requires that the entire continental, national, or regional electricity demand is satisfied annually with local electricity generation from wind, sun, biomass, and water. Within a year, electricity can be traded freely, as long as net annual imports reach 0. Thus, supply scales demand net self-sufficiency.

Balancing scale is always equal to or larger than supply scale. It defines the area in which electricity can be traded within a year. Balancing scale, therefore, demands full self-sufficiency. We model the balancing scale by prohibiting electricity transmission between units on that scale. For national and regional scales this means that no electricity can flow between countries or regions. For the continental scale, this is given inherently by the scope of our study area.

Within a system with national supply and continental balancing, for example, all European countries are net self-sufficient and generate sufficient amounts of renewable electricity to cover national demands. Countries can trade electricity with all other countries in Europe to balance renewable fluctuations. The continent is fully self-sufficient.

### Electrical Load

We determine electrical load profiles for each regional administrative unit following the method described in Tröndle et al.^6^ First, we derive the location and annual demand of industrial facilities with highest electricity demand in Europe from emission data of the European Emission Trading Scheme.^36^ We assume industrial load to be nearly constant and thus derive flat industry profiles for each regional administrative unit.

Second, we use measured national load profiles of 2016^37^ (for Albania no 2016 data are available, and thus, we use 2017 data) and subtract industrial demand to retrieve national profiles of residential and commercial load. We then assume residential and commercial load to be spatially distributed proportional to population counts. Using the Global Human Settlement Population Grid with a resolution of 250 m^38^ we allocate residential and commercial load to regional administrative units (see Experimental Procedure S6 for a discussion of the impact of this estimation method on our results).

Finally, we sum the two industrial and residential time series in each administrative unit to retrieve electricity load profiles at each network node.

### Photovoltaics

PVs can be built at each network node. For each administrative unit, we first determine the maximum amount of capacity that can be deployed. Then, we determine the capacity factor time series that maps from installed capacity to electricity generation at each point in time.

Our model differentiates between PV deployed on open fields and on roof tops and uses geospatial data with a 10 arcsecond resolution. We allow open-field PV to be built on areas of bare land^39^ or open vegetation^39^ that are not environmentally protected,^40^ not inhabited (i.e., <1% of the grid cell are buildings or urban greens according to Ferri et al.^41^), and whose average slope^42^^,^^43^ is 10° at maximum.^44^ We assume a capacity density of 80 W/m^2^ to derive the maximum amount of installable open-field PV capacity for all regional administrative units.

To determine the maximum installable capacity of roof-mounted PV, we consider inhabited areas only (i.e., ≥1% of the grid cell are buildings or urban green areas according to Ferri et al.^41^). Within those grid cells, we use building footprints from Ferri et al.^41^ as a proxy for the amount of available roof tops. Using the high-resolution Sonnendach.ch dataset for Switzerland,^45^ we find that within Switzerland, the ratio between building footprints from Ferri et al.^41^ and rooftops available for PV deployment is 0.56. Due to the lack of comparable data for other countries, we apply this ratio for all of Europe to derive the maximum amount of roof space available for PV. We further differentiate between roof space on flat roofs and on tilted roofs based on the ratio from Swiss Federal Office of Energy^45^ and assume capacity densities of 160 W/m^2^ for tilted roofs and 80 W/m^2^ for flat roofs.

We derive capacity factor time series for roof-mounted and open-field PV on a regular grid with 50 km edge length, resulting in around 2,700 time series covering the study area. We assume a performance ratio of 90% and simulate the time series using bias-corrected data from Renewables.ninja.^46^ For roof-mounted PV, because tilt and orientation of tilted roofs have a significant impact on capacity factors, we model 16 different deployment situations covering roofs facing east, south, west, and north, with tilts between 18° and 43°. We calculate a weighted average from the resulting 16 time series based on the distribution of roofs from Swiss Federal Office of Energy^45^ to derive a single time series for roof-mounted PV for each 50 km grid cell. For open-field PV we optimize the tilt based on location,^47^ so each 50 km grid cell has a single time series. By computing the weighted spatial average across grid cells whose centroid lies within a given administrative unit, we finally compute a single open-field PV and a single roof-mounted PV time series for each administrative unit.

### Wind On- and Offshore

Onshore and offshore wind capacities can be deployed at each network node, and we apply a method similar to the one for PVs to derive their maximum amount of installable capacities and their capacity factor time series.

We use geospatial data with 10 arcsecond resolution to derive the maximum amount of installable wind power capacities. We allow onshore wind farms to be built on areas with farmland, forests, open vegetation, and bare land^39^ that are not environmentally protected,^40^ not inhabited (i.e., <1% of the grid cell are buildings or urban greens according to Ferri et al.^41^), and whose average slope^42^^,^^43^ is 20° at maximum.^4^ We allow offshore wind farms to be built in offshore areas within Exclusive Economic Zones^48^ with water depths^49^ not below 50 m and that are not environmentally protected.^40^ We assume capacity densities of 8 and 15 W/m^2^^50^ for onshore and offshore wind. Where land is available for onshore wind farms and open-field PV, either technology or a mix of both technologies can be used. We allocate the installable offshore capacities to those administrative units that share a coast with the Exclusive Economic Zone, and where there is more than one region, we allocate the capacities proportional to the length of the shared coast. We do not explicitly model the transmission network expansion needed to connect offshore farms.

We derive capacity factor time series for on- and offshore wind on the same 50 km^2^ grid as we do for PV, resulting in around 2,700 onshore grid cells and around 2,800 offshore grid cells. We again use bias-corrected data from Renewables.ninja^51^ to simulate wind generation at each grid cell, assume capacity factors to be constant within the cell, and generate a spatially weighted average to generate a capacity factor time series for each regional administrative unit.

### Hydro Run of River and Reservoirs

We assume hydro run-of-river and hydro reservoir potentials to be largely tapped today^52^ with almost no expansion potential. Thus, for hydro generation capacities we allow not more than today’s capacities.

We derive the location and installed power and storage capacities of hydro stations in Europe today from the JRC Hydro Power Database.^53^ Where no storage capacity of hydro reservoirs is available, we use the median national ratio of power to storage capacity, and if that is not available, we use the median Europe-wide ratio of power to storage capacity.

To create power generation time series for each station, we use a two-stage approach. First, we derive water inflow time series for each station using an approach based on ERA5 runoff data^54^ and hydrological basins^55^ described and validated for China in.^56^ We use Atlite^57^ to first determine all basins upstream of the hydropower station to be able to sum all upstream runoff while assuming a water flow speed of 1 m/s.

Second, we apply bias correction factors based on annual generation necessary for this method to represent the actual magnitude of the inflow and thus accurately model power generation. As we do not have data per station, we use national generation data from IRENA.^58^ For hydro run-of-river plants we assume constant annual capacity factors within each country, which allows us to estimate the annual generation per plant. We use this estimation to derive electricity generation time series for each plant by scaling and capping the water inflow time series such that they sum to the annual generation without ever exceeding power capacities of the stations. For hydro reservoirs, we additionally assume they never need to spill water, i.e., their storage capacity is sufficient to use all inflowing water. We then scale the water inflow time series in such a way that they sum to the annual generation of the stations.

Using location data of each plant, we sum up time series as well as power and storage capacities per regional administrative unit. Our total resulting capacities are 36 GW for run of river and 103 GW/97 TWh for reservoirs.

### Bioenergy

We use estimations of biomass potentials for the year 2020 and reference assumptions taken from Ruiz Castello et al.,^59^ but we assume no dedicated farming for energy crops and thus consider residuals and wastes only. The potentials sum to an European potential of 2,400 TWh/year primary energy in our entire study area, which is used in none of our cases by more than 50% (see Figure S8). The data are given as national aggregates, and we use national shares of farmland,^39^ national shares of forests,^39^ and national shares of population^38^ as proxies to derive proportionally allocated potentials per regional administrative unit. Table S4 lists all feedstocks we consider together with the allocation proxy we use.

We do not discriminate between materials stemming from different feedstocks and assume an efficiency of 45% for the combustion of all biomass.^38^ We furthermore assume that sufficient levels of storage options are available such that there is no further temporal restriction on the combustion other than the annual potential. This flexibility allows to use bioenergy combustion to balance seasonal fluctuations of solar power for example, which is used in some regions of our study area (see Figure S9).

### Pumped Storage Hydro

Similar to hydro run-of-river and hydro reservoir capacities, we assume pumped storage hydro capacities in Europe to be largely tapped^52^ and do not allow for capacity expansion. Thus, we deploy not more than today’s pumped hydropower and storage capacities. We assume a round-trip electricity efficiency of 78%.^60^

To determine location, power, and storage capacity of each pumped hydro station in Europe today, we also use the JRC Hydro Power Database.^53^ Where storage capacities are missing, we employ the same method as for hydro reservoirs: we assume national median ratios of power to storage capacity for all stations with missing storage capacity; and where this is not available, we assume Europe-wide median ratios of power to storage capacity. The storage capacities from the JRC Hydro Power Database sum up to more than 10 TWh, which is an order of magnitude above the 1.3 TWh reported by Geth et al.^61^ To ensure that we do not overestimate the pumped storage potential, we, therefore, scale storage capacities to match national data reported by Geth et al.^61^ Using location data of each station, we then sum all power and storage capacities within regional administrative units to form a single pumped hydro capacity per unit.

### Short-Term and Long-Term Storage

We assume that short-term and long-term storage capacities can be deployed in all regional administrative units. We model short-term storage as Lithium-ion batteries and assume long-term storage is provided by hydrogen stored in overground steel tanks,^62^ as they are likely to become the dominant technology in their respective applications.^60^ The models are based on two technical parameters: the ratio between power and storage capacity and the round-trip efficiency. Short-term storage is constrained to a maximum capacity of 4 h of full power, while long-term storage has a minimum of 4-h capacity at full power. We assume 86% of round-trip efficiency for short-term and 40% for long-term storage.

Additionally, we assume that power and storage capacities can be expanded independently, constrained only by the above-mentioned minimum and maximum storage capacities.

### Insufficient Potentials

In some regions, local technical potential for renewable electricity is not high enough to satisfy local electricity demand.^6^ This is problematic in system layouts in which regions strive for self-sufficiency. To provide sufficient electricity supply in these regions, we connect them with a neighboring or the encompassing region: Vienna with Lower Austria, Brussels with Flanders, Berlin with Brandenburg, Oslo with Akershus, and Basel-City with Basel-Country. For the regional-scale system, but also for continental- and national-scale systems with regional self-sufficiency, we therefore require self-sufficiency of each combined region in these five corner cases.

### Technology Cost

We assess the long-term (quasi steady-state) cost of electricity supply. We aim neither to determine the cost of a transition to a future system nor to consider disruptive developments on the global market for supply and storage technologies. Thus, our costs are based on expected learning rates and the assumption that renewable generation and electricity storage technologies will have been deployed at cumulative capacities consistent with our study. Cost estimates for the year 2050 are primarily from Robert et al.^63^ for supply and transmission technologies, from Schmidt et al.^60^ for storage technologies, and from Ruiz Castello et al.^59^ for fuel cost of bioenergy. See Table S5 for an overview of all cost assumptions.

Technology cost is modeled as the sum of overnight capacity cost, annual maintenance cost based on installed capacity, and variable cost per unit of generated electricity. For solar and wind, we assume a small variable cost of 0.1 €ct/kWh to encourage curtailment whenever generation potential is higher than demand and storage capacities. We subtract these variable costs from the fixed operation and maintenance costs based on average capacity factors, so that they do not increase the overall cost of solar or wind technologies. For all hydropower technologies, we consider annual maintenance and variable cost only, since we assume that maximum capacities are already built today, so overnight cost of hydropower has no impact on our results.

Technology lifetime and cost of capital are used to derive annuities for each technology. We assume cost of capital to be 7.3% for all technologies and all locations based on historic average cost of capital for OECD countries.^64^ Some recent literature suggests cost of capital are likely specific to technology^64^^,^^65^ and location,^64^^,^^66^ but we consider the data available so far too sparse to provide a solid basis on which to model this.

### Sensitivity to Meteorological Conditions

While we use only a single year of meteorological conditions, 2016, in the analysis of system layouts described in the article, we use 10 years, 2007–2016, to analyze the sensitivity of our results to meteorological conditions impacting generation from wind and solar power. We keep all other factors, including time series for electricity demand and hydropower, fixed. We re-run the model with the full 10 years of data considered for the optimization. In this way, we are not assessing the variability between meteorological years, but we are assessing how much the result changes when considering a wider range of meteorological conditions.

We are interested in the sensitivity of one of the main outputs of our study: relative total system cost of the national-scale system using the continental-scale system as a baseline. Because computational requirements to solve a model with regional spatial resolution and temporal resolution of 4 h for 10 years are too high, we perform this sensitivity analysis using a model with national spatial resolution while keeping temporal resolution the same. Comparing the results between national and regional resolution for the case with only 1 year of meteorological conditions, we find a difference of 8% for the relative cost of the national-scale system.

The additional cost of the national-scale system compared with the continental-scale system, however, is unaffected by the longer time duration: the difference to the case with only 1 year is negligible (< 1‰). This is not to say that cost and design of the electricity system is not sensitive to meteorological conditions. In fact, we find that total system cost is generally slightly higher, and more wind and bioenergy capacities are deployed in exchange of solar capacities. However, large-scale and small-scale systems are impacted similarly, and so the difference between both is unaffected by the longer time duration of considered meteorological conditions. These results justify the use of only a single meteorological year.

### Sensitivity to Technology Cost

We furthermore assess the uncertainty of our results stemming from uncertainty of technology cost. While we do know current cost and that it is likely to fall with deployment due to learning effects, we do not know exact future cost with certainty. This uncertainty stems primarily from two sources. We do not know the deployment rates of renewable technologies, and we do not know how much cost will fall with deployment. In our analysis, we are assuming that renewable technologies are heavily deployed, so we focus on the second uncertainty: the relationship between deployment and cost reductions. Since we perform cost minimization, the absolute total system cost of any assessed electricity system layout can be sensitive to the cost of its constituent technologies, as shown for example in Moret et al.^67^

We assess the sensitivity of differences in system cost and technology deployment between large and small-scale system layouts. We consider as uncertain parameters the cost of ten different technologies, the weighted cost of capital, and the availability of biomass for combustion. Following a maximum entropy approach, we model their uncertainty with uniform distributions over ranges taken from the literature (see Table S1). We perform a global sensitivity analysis of system cost and several other model outputs in this twelve-dimensional space. This allows us to derive the distribution of each model output, and it allows us to derive total and first-order Sobol’ indices. The Sobol’ indices determine the share of the variance of each output that is explained by the uncertainty of each input. Building on this, we use the indices to compare the relative importance of all input parameters for the uncertainty of each output.

To derive the output distribution and Sobol’ indices, we need to let parametric uncertainty propagate into and through the model. In a classical Monte Carlo simulation, the input distributions are sampled many times to derive samples of the output. Because of the high computational requirements, in particular the time our model takes to run, this approach would be prohibitive for our study. Thus, we employ a method described in Sudret^68^ and Le Gratiet^69^ to perform a polynomial chaos expansion of our original model to derive a surrogate model. We use the MATLAB package UQLab.^70^ From this surrogate model, Sobol’ indices can be determined analytically, and the distribution of the outputs can be derived using Monte Carlo sampling. We derive the surrogate model by sampling 150 times from the input parameters using maximin Euclidean-distance-optimized Latin Hypercube Sampling and by running continental-, national-, and regional-scale models each once for each input parameter vector. Due to the high computational requirements of running national- and continental-scale models, we perform these runs on a spatial resolution with low fidelity in which each country represents one transmission grid node. To remove the biases these low fidelity model runs introduce, we perform 10 additional runs on the original, high-fidelity resolution and use a multi-fidelity approach^71^ to retrieve a single surrogate model for continental and national scales. The estimated cross-validation error of the surrogate model is below 5%, and thus, we deem the surrogate sufficiently accurate^69^ to derive total- and first-order Sobol’ indices.

## Supplemental Information

Document S1. Supplemental Experimental Procedures, Figures S1–S9, Tables S1–S5, and Supplemental References

Document S2. Article plus Supplemental Information
