# Supplementary material for: Trade-Offs between Geographic Scale, Cost, and Infrastructure Requirements for Fully Renewable Electricity in Europe
Source: Joule. 2020 Sep 16;4(9):1929–48. doi: 10.1016/j.joule.2020.07.018 (PMC7498190; doi:10.1016/j.joule.2020.07.018)
Supplement: Document S1. Supplemental Experimental Procedures, Figures S1–S9, Tables S1–S5, and Supplemental References [file mmc1.pdf]

JOUL, Volume *4*

Supplemental Information

Trade-Offs between Geographic Scale, Cost, and Infrastructure Requirements for Fully Renewable Electricity in Europe

Tim Tröndle, Johan Lilliestam, Stefano Marelli, and Stefan Pfenninger

Figure S1: Transmission network

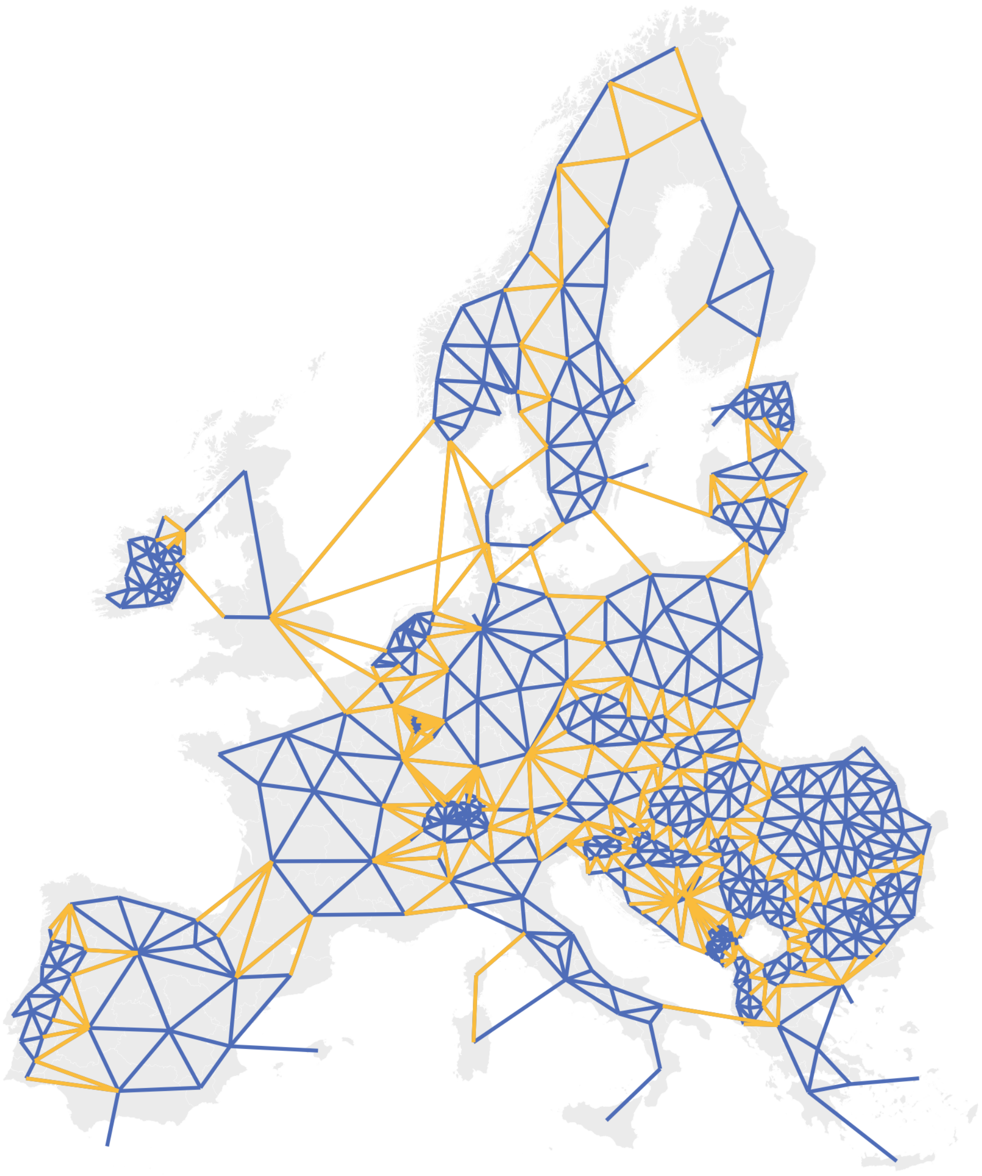

Figure S1: Possible locations of transmission capacities. All lines visualise connections between two regions that can hold transmission capacities. International connections are coloured yellow, all others are coloured blue. The amount of capacities installed on these connections is an output of the optimisation and depends on the considered case.

Figure S2: System cost in cases including net imports

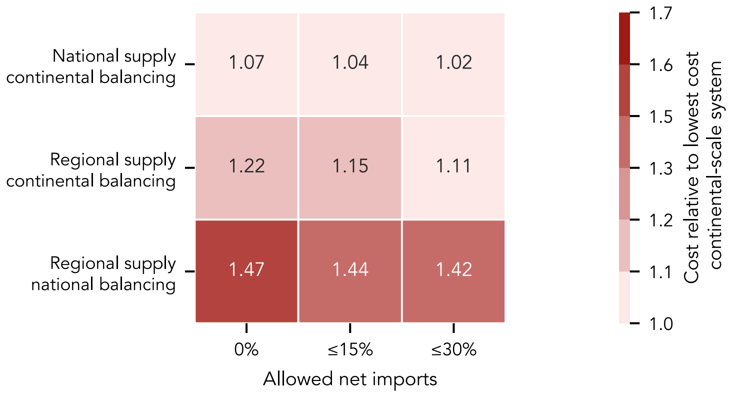

Figure S2: System cost of nine electricity systems in Europe with national or regional net supply, and continental or national balancing, relative to lowest cost, continental-scale system. Cost of variations of three cases from Figure 1 in which net imports into the national or regional supply area are allowed to certain degrees (x-axis), from no net imports (0%, corresponds to cases from Figure 1) to imports covering up to 30% of national or regional electricity demand. Cost are relative to the entirely continental case in Figure 1.

Figure S3: System composition of all cases

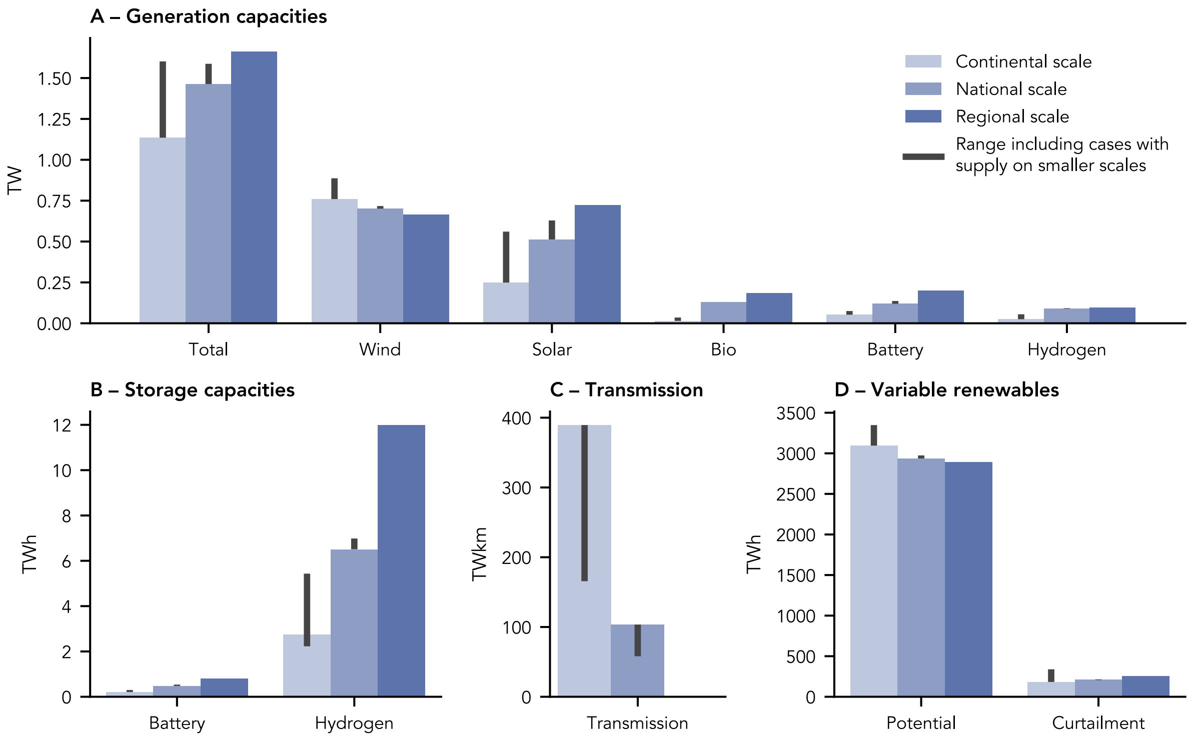

Figure S3: Cost-optimised technology mixes for all cases. A,B,C,D,, Europe-wide installed generation capacities (**A**), storage capacities (**B**), transmission capacities (**C**), and potential and curtailed generation from variable renewable sources (**D**) for entirely continental-, national-, and regional-scale electricity systems with minimal system cost. The thin bars indicate the range of values including cases with supply on smaller scales. See Tables S2 and S3 for numerical results of all cases. **A**, The total excludes all storage capacities but includes capacities of hydropower at or below today's levels (36 GW run of river, 103 GW reservoirs). **B**, Europe-wide installed storage capacities for battery and hydrogen storage. Up to 97 TWh from hydro reservoirs and 1.3 TWh from pumped hydro storage are not shown. **C**, Europe-wide installed electricity transmission capacities, ignoring transmission capacities within regions. **D**, Europe-wide potential generation and curtailed generation from the variable renewable sources solar, wind and hydro.

Figure S4: Generation shares

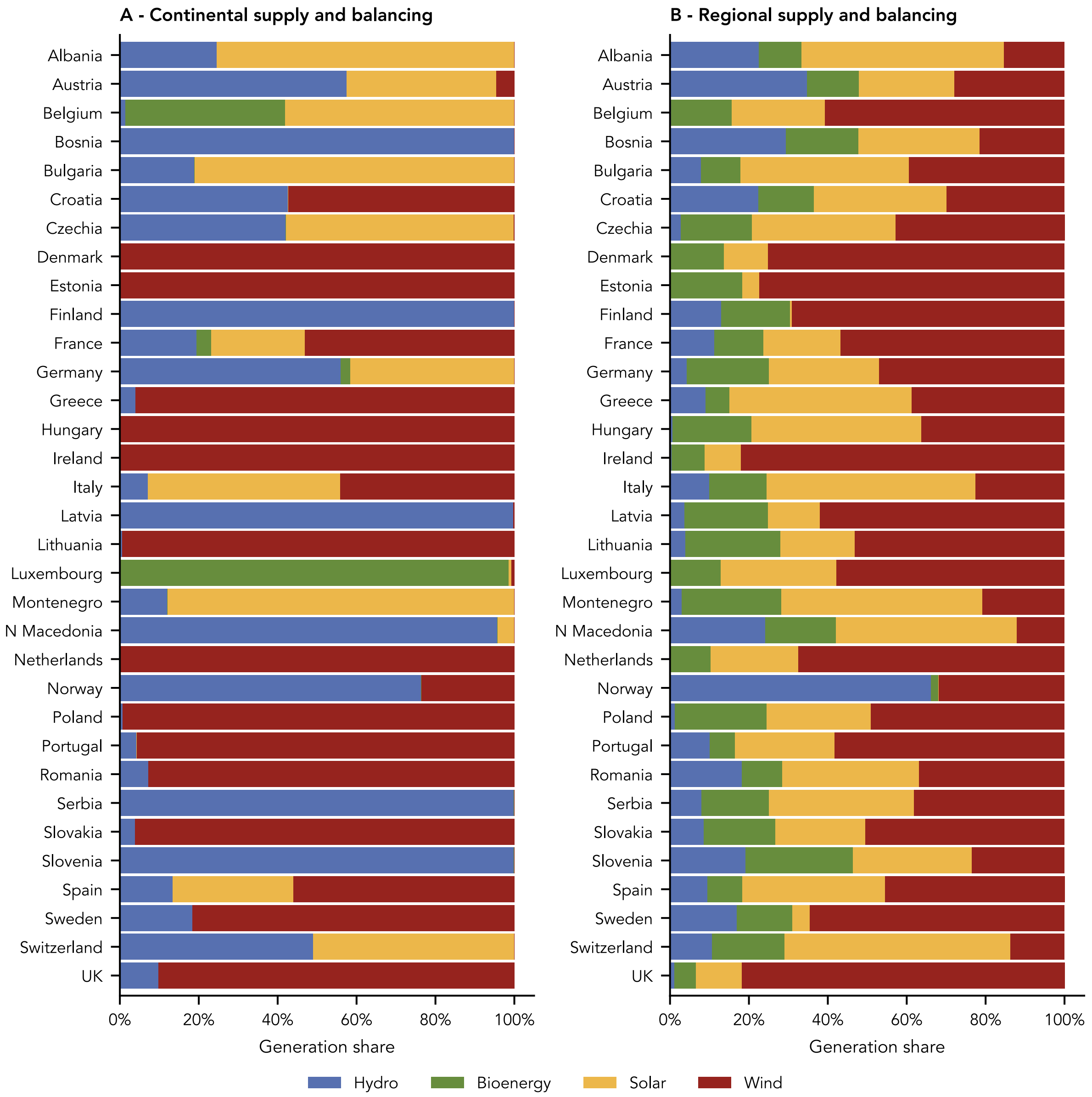

Figure S4: Technology generation shares for all countries. A, Generation shares when supply and balancing are continental. **B,** Generation shares when supply and balancing are regional.

Figure S5: Total Sobol' indices

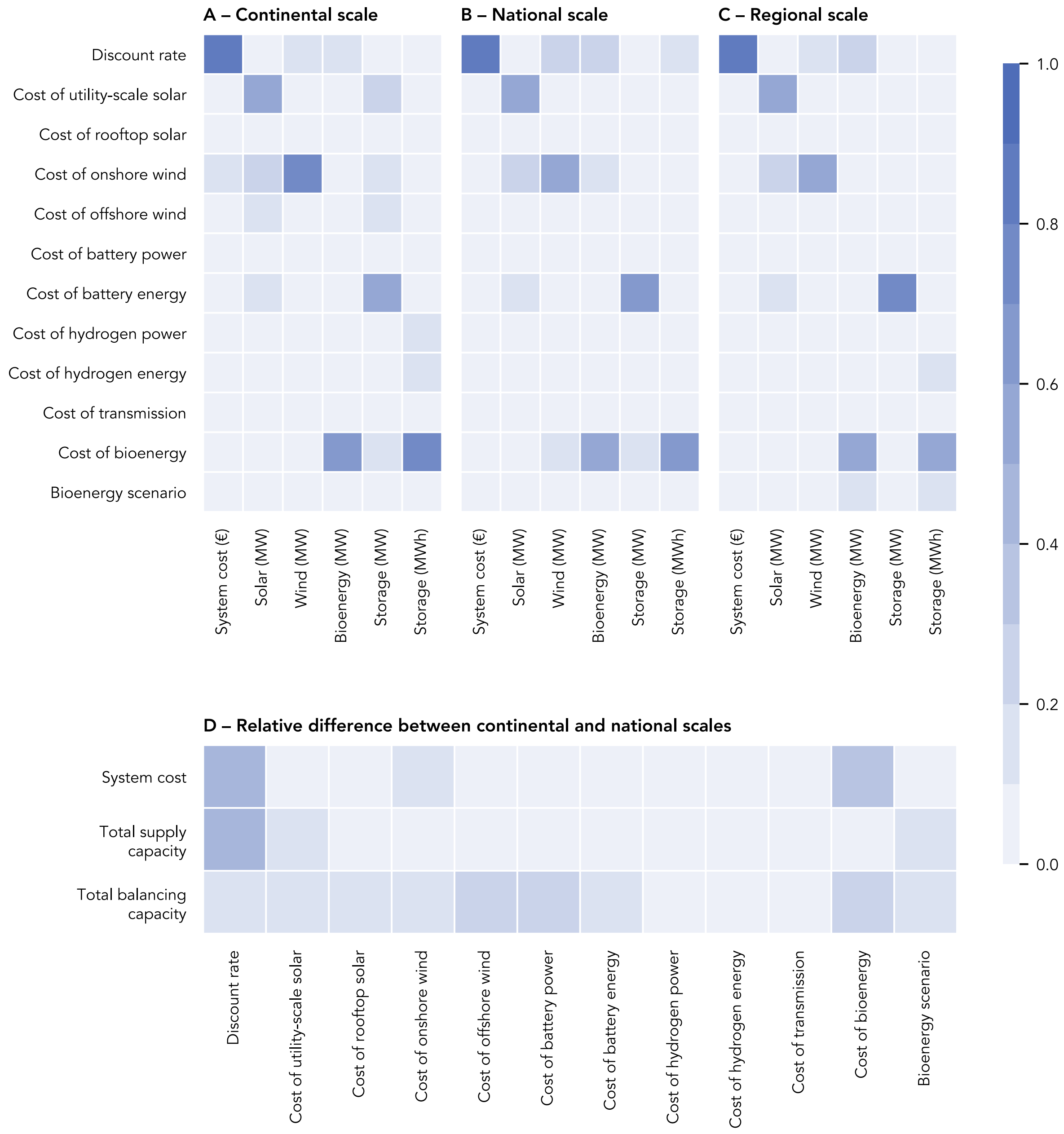

Figure S5: Total Sobol' indices for combinations of all considered input uncertainties and selected model outputs of entirely continental-, national-, and regional-scale electricity systems. A,B,C, Sobol' indices of input parameters considering total system cost and total installed capacities (x-axis) of the continental- (**A**), national- (**B**), and regional-scale (**C**) systems. The y-axis shows the twelve input parameters included in the uncertainty analysis. **D,** Sobol' indices of input parameters considering difference in system cost between the continental- and national-scale systems. The x-axis shows the twelve input parameters included in the uncertainty analysis. The y-axis shows the model-wide result variables for which continental to national scale differences are compared.

Figure S6: First-order Sobol' indices

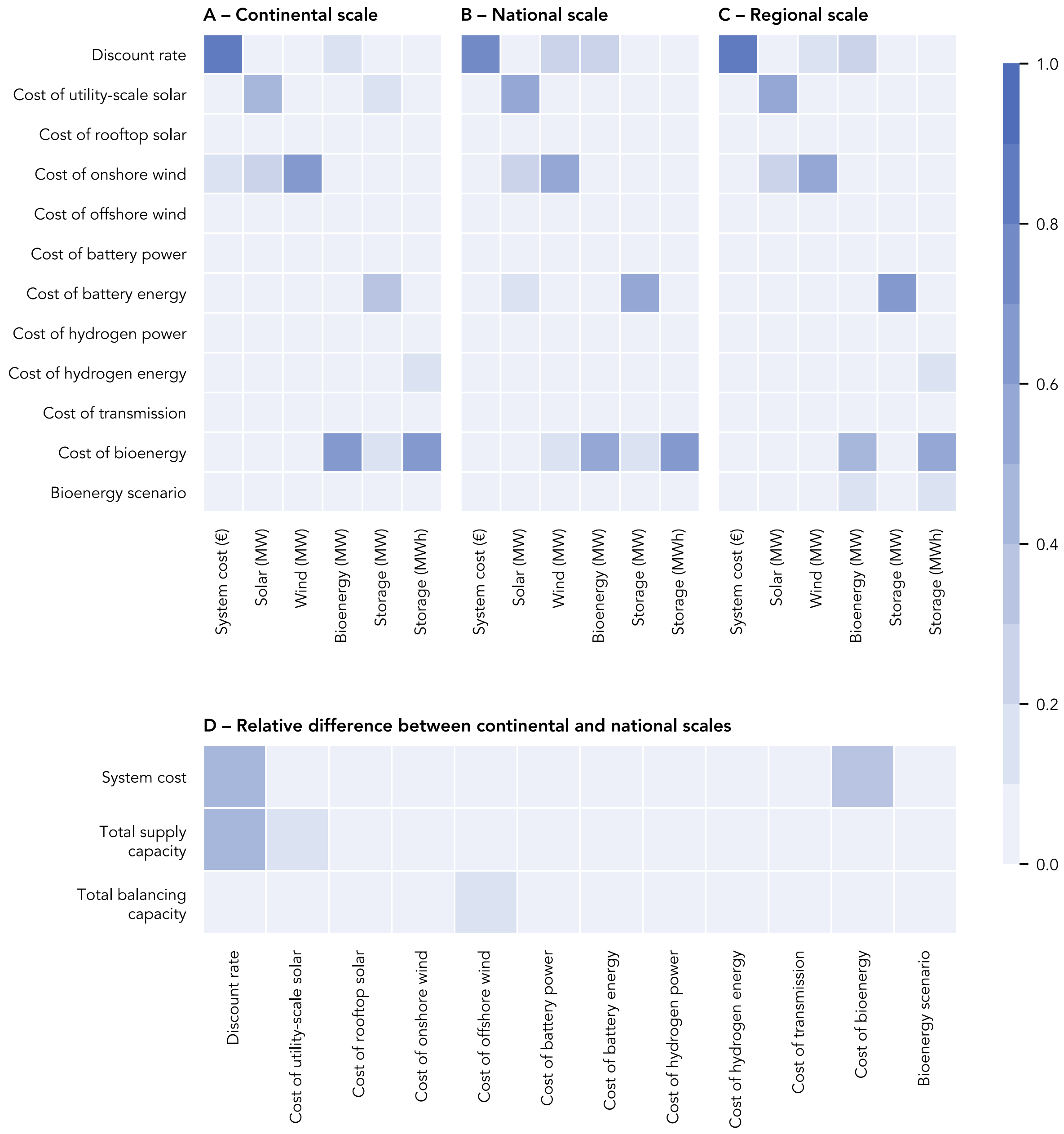

Figure S6: First-order Sobol' indices for combinations of all considered input uncertainties and selected model outputs of entirely continental-, national-, and regional-scale electricity systems. A,B,C, Sobol' indices of input parameters considering total system cost and total installed capacities (x-axis) of the continental- (**A**), national- (**B**), and regional-scale (**C**) systems. The y-axis shows the twelve input parameters included in the uncertainty analysis. **D,** Sobol' indices of input parameters considering difference in system cost between the continental- and national-scale systems. The x-axis shows the twelve input parameters included in the uncertainty analysis. The y-axis shows the model-wide result variables for which continental to national scale differences are compared.

Figure S7: Difference Sobol' indices

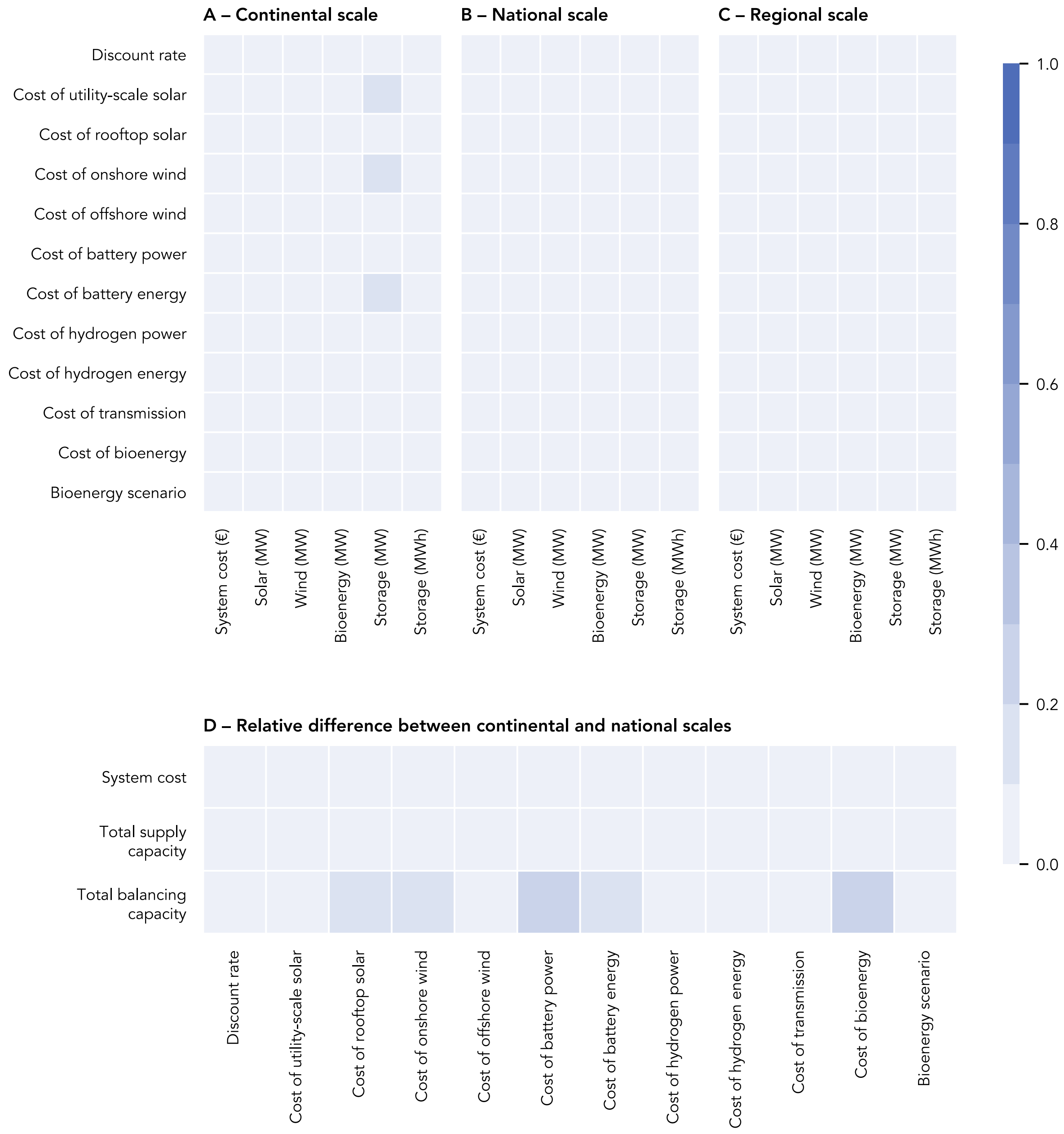

Figure S7: Total minus first-order Sobol' indices for combinations of all considered input uncertainties and selected model outputs of entirely continental-, national-, and regional-scale electricity systems. A,B,C, Sobol' indices of input parameters considering total system cost and total installed capacities (x-axis) of the continental- (**A**), national- (**B**), and regional-scale (**C**) systems. The y-axis shows the twelve input parameters included in the uncertainty analysis. **D,** Sobol' indices of input parameters considering difference in system cost between the continental- and national-scale systems. The x-axis shows the twelve input parameters included in the uncertainty analysis. The y-axis shows the model-wide result variables for which continental to national scale differences are compared.

Figure S8: Use of bioenergy potentials

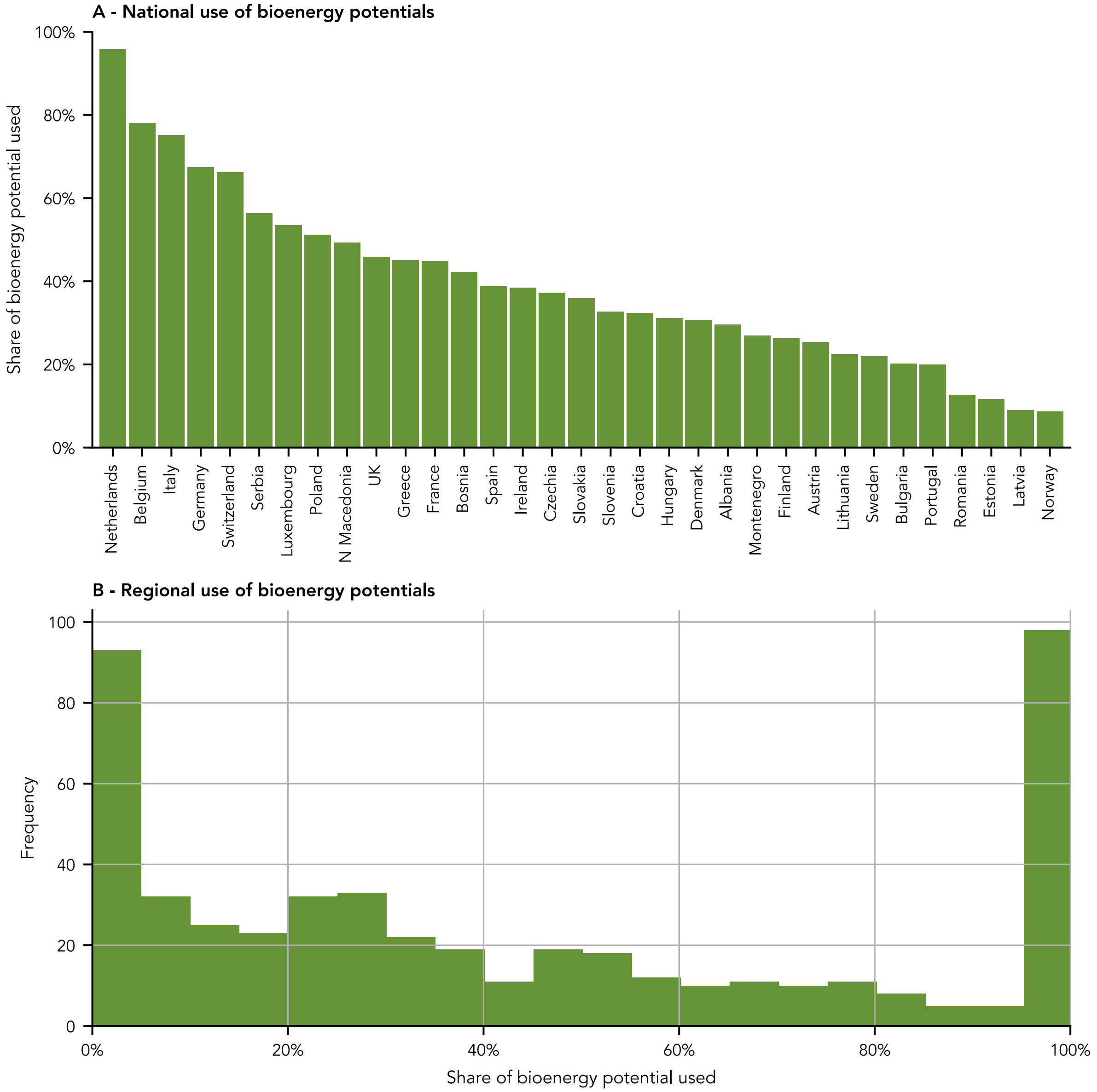

Figure S8: Use of bioenergy potentials. All use data from the case with highest levels of deployed bioenergy capacity in which supply and balancing are regional. Potentials are based on estimations of available residual material[1] and sum to 2400 TWh/yr Europe-wide (1100 TWh/yr usable electricity) for the year 2020 and reference assumptions. **A,** Use of national potentials. **B,** Use of potentials in 497 subnational regions.

Figure S9: Distribution of time series across regions

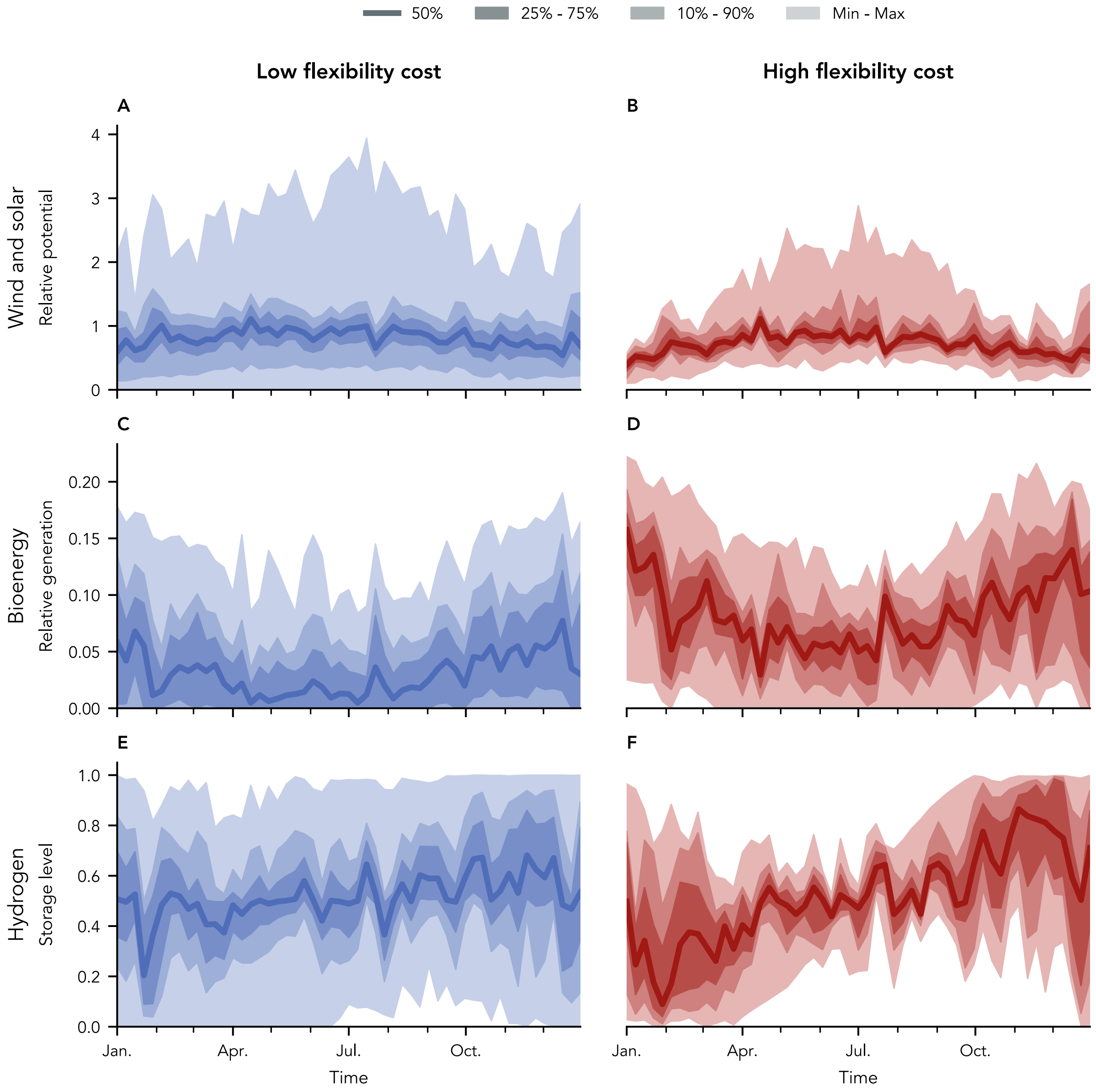

Figure S9: Distribution of time series for all regions of the entirely regional-scale electricity system. **A,B,C,D,E,F,** Distribution of time series for all regions with low flexibility cost (combined cost of bioenergy, hydrogen, and battery storage) (**A,B,C**) and all regions whose flexibility cost lies within the highest decile (**D,E,F**). **A,B,** Combined wind and solar weekly generation potential time series relative to local demand. Seasonal fluctuations are more pronounced for the case with higher cost due to higher shares of solar electricity. Regions with high solar shares are often urban regions with low or no wind potential. **C,D,** Weekly generation time series from biomass combustion relative to demand. Generation has a more pronounced seasonality and is generally larger in regions with higher flexibility cost. **E,F,** Weekly time series of hydrogen storage levels relative to installed storage capacity. In regions with higher cost, hydrogen storage is used primarily to balance seasonal fluctuations, instead of balancing fluctuations within weeks or months as it is done for lower cost regions, leading to fewer storage cycles and higher cost.

Table S1: Input parameter uncertainty

Table S1: Uncertain input parameters. For all parameters we assume a uniform distribution.

Name	Description	Min	Max	Unit	Source
Discount rate	System- and sector-wide discount rate of investments.	0.016	0.138	-/yr	Max and min for solar and wind between 2009 and 2017 in ref. 2
Cost of utility-scale solar	Cost of installing photovoltaics capacity on fields.	280000.0	580000.0	EUR/MW	Ref. 3 (Table 7)
Cost of rooftop solar	Cost of installing photovoltaics capacity on rooftops.	760000.0	1000000.0	EUR/MW	Ref. 3 (Table 9)
Cost of onshore wind	Cost of installing onshore wind capacity.	800000.0	1700000.0	EUR/MW	Ref. 3 (Table 4)
Cost of offshore wind	Cost of installing offshore wind capacity.	1790000.0	3270000.0	EUR/MW	Ref. 3 (Table 5)
Cost of battery power	Cost of installing battery power capacity (inverter etc.).	31000.0	141000.0	EUR/MW	Ref. 4
Cost of battery energy	Cost of installing battery energy capacity.	36000.0	166000.0	EUR/MWh	Ref. 4

Name	Description	Min	Max	Unit	Source
Cost of hydrogen power	Cost of installing hydrogen power capacity (fuel cell etc.).	1123000.0	2100000.0	EUR/MW	Ref. 4
Cost of hydrogen energy	Cost of installing hydrogen energy capacity.	6000.0	12000.0	EUR/MWh	Ref. 4
Cost of transmission	Cost of installing net transfer capacity (high voltage ac transmission).	0.7	1.08	EUR/MW/m	Ref. 3 (Table 39)
Cost of bioenergy	Cost of installing biofuel capacities.	1380000.0	3450000.0	EUR/MW	Ref. 3 (Table 48)
Availability and fuel cost of bioenergy	Interpolation between biofuel potential and fuel cost scenarios.	0.0	1.0	•	Ref. 1

Table S2: Generation and storage capacities

Table S2: Installed capacities of photovoltaics (PV), on- and offshore wind, bioenergy, short-term (battery) and long-term (hydrogen) storage, and relative curtailment of solar, wind, and hydropower for all considered cases. Each case additionally contains fixed hydropower capacities on their current locations: 36 GW run of river, 103 GW / 97 TWh reservoirs, and 54 GW / 1.3 TWh pumped hydro storage.

Case	PV (GW)	Wind (GW)	Curtailment (%)	Bioenergy (GW)	Battery (GW)	Battery (GWh)	Hydrogen (GW)	Hydrogen (GWh)
Entirely continental case	249	760	6	15	54	218	26	2754
Entirely national case	513	701	7	131	119	477	89	6508
Entirely regional case	723	665	9	184	202	807	97	11988
Continental scale balancing, national scale supply and 0% net imports	309	843	10	35	52	210	25	2228
Continental scale balancing, national scale supply and 15% net imports	288	814	9	35	52	210	20	1865
Continental scale balancing, national scale supply and 30% net imports	257	803	8	30	49	194	21	2189
Continental scale balancing, regional scale supply and 0% net imports	561	887	10	32	75	300	55	5433
Continental scale balancing, regional scale supply and 15% net imports	492	842	10	42	67	267	33	3104
Continental scale balancing, regional scale supply and 30% net imports	430	813	9	45	61	243	19	1802
National scale balancing, regional scale supply and 0% net imports	629	717	7	131	136	537	93	6984

Case	PV (GW)	Wind (GW)	Cur- tail- ment (%)	Bioen- ergy (GW)	Bat- tery (GW)	Bat- tery (GWh)	Hy- dro- gen (GW)	Hydro- gen (GWh)
National scale balancing, regional scale supply and 15% net imports	582	701	7	134	126	503	90	6597
National scale balancing, regional scale supply and 30% net imports	560	692	7	136	125	501	87	6385

Table S3: Transmission capacities

Table S3: Installed transmission grid capacity, gross physical electricity flow crossing country borders, and net electricity flow imported by all countries for all cases.

Case	Transmission (TW km)	National import gross (TWh)	National import net (TWh)
Entirely continental case	389	2981	1463
Entirely national case	103	0	0
Entirely regional case	0	0	0
Continental scale balancing, national scale supply and 0% net imports	267	1631	26
Continental scale balancing, national scale supply and 15% net imports	288	1785	388
Continental scale balancing, national scale supply and 30% net imports	316	2098	748
Continental scale balancing, regional scale supply and 0% net imports	166	1308	46
Continental scale balancing, regional scale supply and 15% net imports	195	1467	349
Continental scale balancing, regional scale supply and 30% net imports	237	1747	651
National scale balancing, regional scale supply and 0% net imports	58	0	0
National scale balancing, regional scale supply and 15% net imports	67	0	0
National scale balancing, regional scale supply and 30% net imports	78	0	0

Table S4: Biomass feedstocks

Table S4: Biomass feedstocks we consider, together with the proxy we use to derive regional from national values.

Feedstock	Proxy
Manure biomass	Farmland
Primary agricultural residues	Farmland
Roundwood fuelwood	Forests
Roundwood Chips & Pellets	Forests
Forestry energy residue	Forests
Secondary forestry residues – woodchips	Forests
Secondary Forestry residues – sawdust	Forests
Forestry residues from landscape care	Forests
Municipal waste	Population
Sludge	Population
Landscape care residues	Population

Table S5: Technology cost assumptions

Table S5: Assumptions on technology cost. ^AC transmission overnight cost is given in €/kW/1000km

Technology	Overnight cost (€/kW)	Overnight cost (€/kWh)	Annual cost (€/kW/yr)	Variable cost (€ct/kWh)	Lifetime (yr)	Source
Utility-scale PV	520	0	8	0	25	Ref. 3 Table 7
Rooftop PV	880	0	16	0	25	Ref. 3 Table 9
Onshore wind	1100	0	16	0	25	Ref. 3 Table 4
Offshore wind	2280	0	49	0	30	Ref. 3 Table 5
Biofuel	2300	0	94	6	20	Ref. 3 Table 48, ref. 1
Hydropower run of river	0	0	169	1	60	Ref. 3 Table 14
Hydropower with reservoir	0	0	101	1	60	Ref. 3 Table 12
Pumped hydro storage	0	0	7	0	55	Ref. 4
Short term storage	86	101	1	0	10	Ref. 4
Long term storage	1612	9	14	0	15	Ref. 4

Techno-logy	Overnight cost (€/kW)	Overnight cost (€/kWh)	Annual cost (€/kW/yr)	Variable cost (€ct/kWh)	Lifetime (yr)	Source
AC trans-mission^	900	0	14	0	60	Ref. 3 Table 39

Supplemental Experimental Procedures

Procedure S1: Impact of net imports into supply area on cost

To confirm the inferior impact of supply options on total cost, we assess the cost of nine further cases, in which we relax the supply scale by permitting net imports to satisfy national or regional electricity demand. This relaxation has a small impact on cost (Figure S1), with 10 percentage points or less cost reduction between net self-sufficiency (0 net imports, cases from Figure 1) and allowing up to 30% net imports. This reinforces the finding from Figure 1: geographic scale has a particularly large impact on cost because of the possibilities for balancing, not mainly because of supply options.

Procedure S2: Today's transmission capacities

To determine today's transmission capacities, we use an approach described in ref. 5 together with data from the same publication. Ref. 5 uses an extended version of the GridKit tool[6] to find the location, voltage level, and number of circuits of all transmission lines in Europe. They estimate the transmission capacity per line based on the voltage level. In our study area, this leads to ~190 GW of international (cross-border) transmission capacity, ~1,600 GW of interregional transmission capacity, and a total of ~340 TWkm of transmission capacity.

To make the total number comparable to results from our model, we adjust it in the following way: We start by determining the transmission capacity between each pair of regions (in GW). We then ignore the actual lengths of the lines and instead map transmission capacities to transmission lines of our model, which connect centroids of all regions (Figure S1). Using this approach, we estimate today's interregional transmission capacity to be 215 TWkm.

Procedure S3: Effect of hydropower model choices on our results

The way in which we model hydropower generation in Europe leads to peculiarities in results on the regional scale. Here, we find the lowest system cost in regions with large hydropower installations. These cost peculiarities are consequences of our design choice: we assume they are amortised. In the following, we justify this model design choice and discuss its relevance for our results.

We assume hydropower capacities are amortised to avoid the need to model overnight cost. Overnight cost of hydropower capacities can vary strongly between projects and are thus difficult to model. Ignoring overnight cost can lead to low levelised cost of electricity in regions with large hydropower capacities. Cost is particularly low when dams provide local flexibility and other forms of more expensive flexibility provision can be avoided. Thus, our model choice leads to low cost in some regions and it also leads to slightly optimistic absolute system cost. But because we fix capacities to current levels for all Europe in all cases, the ignored overnight cost imply no benefit to any case and thus do not affect relative cost.

Procedure S4: Effect of model simplifications

There are three aspects which our analysis does not consider and which may impact our findings. Some are likely to increase attractiveness of small-scale systems, others are likely to increase attractiveness of large-scale systems.

Most importantly, we do not consider flexibility from electricity demand or from electrifying the heat and transport sectors. These additional flexibilities may be especially beneficial for smaller-scale systems, whose flexibility options are more limited and expensive. However, large electricity systems can and will benefit as well and our sensitivity analysis shows that cost differences are driven largely by the cost of bioenergy; a technology mostly used to balance seasonal fluctuations of solar generation. Only if additional flexibilities can balance seasonal fluctuations, a significant impact on system cost can be expected. This is likely not the case for demand flexibility[7] or transportation[8], but it may be possible by electrifying the heat sector[8].

Furthermore, we do not consider ancillary services for the distribution and transmission grids. The provision of ancillary services may be easier, i.e. less costly, for system layouts as we found them for smaller electricity systems, because not only generation but also support infrastructure is more homogeneously dispersed and thus able to provide services like frequency control or black-start everywhere. However, there is no reason to believe that this would change system cost and therefore relative system cost significantly[9].

We also do not model the distribution grid in any way. The cost of the distribution grid is likely to be higher for smaller systems where generation is dispersed more strongly with substantial amounts of generation

from roof mounted PV embedded within the distribution grid. However, technical potentials of wind and utility-scale PV are high enough in most regions in Europe so that roof mounted PV is rarely necessary. Thus, cost of the distribution grid may be higher for smaller scales, but only if roof mounted PV is prioritised over utility-scale PV.

Procedure S5: Temporal resolution of the model

We run the model using a temporal resolution of four hours due to high computational requirements stemming from the high spatial resolution. Our model is spatially resolved into 497 nodes, which represent all first-level administrative units in Europe. Together with the 2,190 timesteps of the four hour temporal resolution, this leads to ~1,100,000 pairs of space and time for which the optimisation algorithm must find a solution. In comparison, models with national spatial and hourly temporal resolution, which are typically used in previous studies[10–12], pose a problem consisting of 265,000 pairs of space and time. Due to the polynomial-time complexity of the optimisation algorithm, computation time does not scale linearly with the problem size. In fact, we find that solving a version of our model that is resolved on the national level runs 45 times faster than the original version of our model, although it has only 17 times fewer nodes.

Solving our model requires > 250 GB of internal memory and > 24h of runtime for each of the 12 cases and each of the 20 high-resolution uncertainty runs we perform. This is only possible using a high performance computing cluster. Increasing the problem size by increasing the temporal resolution is challenging. In fact, we are not aware of any study with larger problem size.

The temporal resolution of our model likely impacts our results. As has been shown before, model results can change significantly with lower temporal resolutions[13]. However, the effect can be expected to be not large for resolutions above six hours and for models that contain inter-temporal constraints like ours[13]. We test the impact on our results by using a version of our model that is spatially resolved on the national level and has a temporal resolution of one hour. We find that total generation capacity remains nearly constant, but that total balancing capacity increases slightly (<5%) compared to a model that has a temporal resolution of four hours. This result indicates that a higher temporal resolution would likely support rather than contradict our main finding that the balancing scale drives cost more strongly than the supply scale.

Procedure S6: Effect of disaggregation method of national electricity loads

The regional disaggregation method based on population counts and industry plants that we use yields regional electricity load time series that are likely stronger correlated than in reality. In the following, we discuss the impact this may have on our findings.

Most importantly, the stronger correlation impacts all cases with balancing scale above the regional level in the same way. As long as all regions within a country are connected through transmission lines, relative fluctuations between regions can be compensated through the grid. In all of these cases, we can expect that our model choice leads to a slight over- or underestimation of national transmission capacity but that the bias is similar in all cases.

In the single case with regional-scale balancing, relative fluctuations between regions cannot be compensated through the grid but instead must be handled in each region individually. Here, we can expect that through our method some regions suffer slightly higher cost for balancing, but that other regions in the same country enjoy lower cost of similar levels. The impact for single regions may be noticable, but the impact on total system cost can be considered low.

In summary, there is likely a small impact on the differences between the cases with balancing above the regional level and the case with balancing at the regional level. However, there is no reason to believe that this difference or any other impact on our results is large, as there is no reason to believe that correlations between regions are much lower than the ones resulting from our method.

Supplemental References

1. Ruiz Castello, P., Sgobbi, A., Nijs, W., Thiel, C., Dalla Longa, F., Kober, T., Elbersen, B., and Hengeveld, G. (2015). The JRC-EU-TIMES model. Bioenergy potentials for EU and neighbouring countries.

2. Steffen, B. (2019). Estimating the Cost of Capital for Renewable Energy Projects (Social Science Research Network).

3. JRC (2014). ETRI 2014 – Energy Technology Reference Indicator projections for 2010-2050 (JRC).

4. Schmidt, O., Melchior, S., Hawkes, A., and Staffell, I. (2019). Projecting the Future Levelized Cost of Electricity Storage Technologies. Joule *0*.

5. Hörsch, J., Hofmann, F., Schlachtberger, D., and Brown, T. (2018). PyPSA-Eur: An open optimisation model of the European transmission system. Energy Strategy Reviews *22*, 207–215.

6. Wiegmans, B. (2016). Gridkit: Gridkit 1.0 'For Scientists' (Zenodo).

7. Aryandoust, A., and Lilliestam, J. (2017). The potential and usefulness of demand response to provide electricity system services. Applied Energy *204*, 749–766.

8. Brown, T., Schlachtberger, D., Kies, A., Schramm, S., and Greiner, M. (2018). Synergies of sector coupling and transmission reinforcement in a cost-optimised, highly renewable European energy system. Energy *160*, 720–739.

9. Brown, T.W., Bischof-Niemz, T., Blok, K., Breyer, C., Lund, H., and Mathiesen, B.V. (2018). Response to 'Burden of proof: A comprehensive review of the feasibility of 100% renewable-electricity systems'. Renewable and Sustainable Energy Reviews *92*, 834–847.

10. Gils, H.C., Scholz, Y., Pregger, T., Luca de Tena, D., and Heide, D. (2017). Integrated modelling of variable renewable energy-based power supply in Europe. Energy *123*, 173–188.

11. Child, M., Kemfert, C., Bogdanov, D., and Breyer, C. (2019). Flexible electricity generation, grid exchange and storage for the transition to a 100% renewable energy system in Europe. Renewable Energy *139*, 80–101.

12. Zappa, W., Junginger, M., and van den Broek, M. (2019). Is a 100% renewable European power system feasible by 2050? Applied Energy *233-234*, 1027–1050.

13. Pfenninger, S. (2017). Dealing with multiple decades of hourly wind and PV time series in energy models: A comparison of methods to reduce time resolution and the planning implications of inter-annual variability. Applied Energy *197*, 1–13.
